# Maternal and neonatal outcomes and clinical laboratory testing of pregnant women with COVID-19 during the BA.5.2/BF.7 surge

**DOI:** 10.1080/21505594.2024.2360130

**Published:** 2024-05-27

**Authors:** Jiali Cao, Zehong Huang, Jing Zeng, Jumei Liu, Weilun Zuo, Zhiying Su, Yujuan Chen, Weiwei Yu, Huiming Ye

**Affiliations:** a Department of Laboratory Medicine, Fujian Key Clinical Specialty of Laboratory Medicine, Women and Children’s Hospital, School of Medicine, Xiamen University, Xiamen, China; b State Key Laboratory of Vaccines for Infectious Diseases, Xiang An Biomedicine Laboratory, School of Public Health, Xiamen University, Xiamen, China; c School of Pharmacy, Xiamen University, Xiamen, China; d Department of Obstetrics, Women and Children’s Hospital, School of Medicine, Xiamen University, Xiamen, China

**Keywords:** Pregnancy, neonate, COVID-19, Omicron, outcomes

## Abstract

The impact of COVID-19 on pregnant women and newborns continues to be a critical societal concern. However, the majority of research focuses on the disease resulting from the early pandemic variants, without sufficient study on the more recent BA.5.2/BF.7. We retrospectively recruited pregnant women giving birth during the surge of the BA.5.2/BF.7 and analysed the risk impact of COVID-19 on maternal and neonatal outcomes. Furthermore, subjects matched through propensity scores were used for the analysis of clinical laboratory tests. A total of 818 pregnant women were enrolled, among 276 (33.7%) were diagnosed with SARS-CoV-2 during childbirth. COVID-19 significantly increased the risk of a hospital length of stay equal to or greater than seven days and neonatal admission to the neonatal intensive care unit, with an aHR of 2.03 (95% CI, 1.22–3.38) and 1.51 (95% CI, 1.12–2.03), respectively. In the analysis of 462 matched subjects, it was found that subjects infected with SARS-CoV-2 tended slight leucopenia and coagulation abnormalities. We found that during the surge of the BA.5.2/BF.7, COVID-19 increased the risk of maternal and neonatal outcomes among Chinese pregnant women. This finding offers significant insights to guide clinical practices involving pregnant women infected with the recently emerged Omicron subvariants.

## Introduction

Even though the World Health Organization (WHO) declared in early 2023 that COVID-19, which had ravaged the globe for over three years, no longer constituted a “Public Health Emergency of International Concern (PHEIC)” [1,2], the health burden imposed by COVID-19 left a lasting impression with nearly 7 million cumulative deaths, imprinting an unforgettable shadow in the history of human combat against infectious diseases. In the post-pandemic era, the response to COVID-19 has gradually shifted towards high-risk populations. As the high-risk group that nurtures the next generation, the condition of pregnant women after contracting COVID-19 naturally became a primary focus. The teams of Claire [3] and Vivek [4] independently evaluated the mortality outcomes of pregnant women in the United States who were diagnosed with COVID-19 during the early stages of the pandemic. They found that compared to the control group, the mortality rate among pregnant women significantly increased during the pandemic. Similarly, an evaluation by Jose and colleagues of the health outcomes of women diagnosed with COVID-19 in the early Omicron phase (dominated by BA.1 and BA.2) revealed an increased risk of severe maternal morbidity and mortality [5]. However, with SARS-CoV-2 continuing to evolve at a high frequency, variants such as BA.5 emerged as the dominant strain globally in the third quarter of 2022 following the early Omicron sublineages BA.1 and BA.2 [6]. Both in vitro and in vivo studies have indicated that BA.5, compared to early Omicron BA.1 and BA.2, exhibits increased pathogenicity and immune evasion capabilities [7–10]. This raises concerns about potential changes in its impact on maternal and neonatal outcomes. However, there is still a paucity of research on the effects of BA.5 and its sub-lineages on maternal and neonatal outcomes. Thus, with the hypothesis that BA.5 and its sub-lineages have a significant impact on maternal and neonatal outcomes, this study focuses on the impact of COVID-19 during the BA.5.2/BF.7 surge of COVID-19 on maternal and neonatal outcomes as well as associated clinical laboratory testing in China from late 2022 to early 2023. It aims to disclose the health risks to pregnant women and newborns imposed by the close sublineages BA.5.2/BF.7 of BA.5, thereby providing a reference for clinical practice.

## Methods

### Study design and data collection

We retrospectively recruited pregnant women giving birth between December 2022, and early January 2023, during the surge of the BA.5.2/BF.7, at the Women and Children’s Hospital, School of Medicine, Xiamen University, Xiamen, China. Pregnant women who gave birth or had pregnancy outcomes during this period with SARS-CoV-2 nucleic acid test results were included in this study.

Participants who met the following exclusion criteria were excluded: 1) Participants opt for abortion due to ectopic pregnancy or congenital malformation; 2) Participants have a previous infected history of SARS-CoV-2. The SARS-CoV-2 positive group included those who tested positive for SARS-CoV-2 nucleic acid before delivery or during admission. Those who had a nucleic acid negative certificate and those who tested negative for SARS-CoV-2 nucleic acid or were asymptomatic during admission were classified as the undiagnosed group. Demographic characteristics, SARS-CoV-2 detection, clinical laboratory, and outcome data of all participants enrolled were extracted from the medical records obtained from the hospital. The study was conducted according to the guidelines of the Declaration of Helsinki and approved by the Institutional Review Board of the Ethics Committee of the Women and Children’s Hospital, School of Medicine, Xiamen University (No. KY-2023-037-K01). Written informed consent was obtained for each participant.

### Definition of high-risk pregnancy

Pregnancy is classified as high-risk when the pregnant woman exhibits the following characteristics: age beyond 15–35, weight beyond 40–85 kg, history of abnormal pregnancy, medical conditions of pregnancy (including diabetes, hypertension, hypothyroidism, and gestational cholestasis), poor delivery conditions (including pelvic abnormalities, multiple births, and abnormal foetal position, macrosomia), cicatricial uterus, excess or low amniotic fluid, in vitro fertilization or embryo transfer, abnormal reproductive structures, congenital anomalies or foetal growth retardation, thalassaemia, and placenta praevia, placenta abruptio or hypofunction.

### Statistical analysis

The estimated adjusted hazard ratio (aHR) was calculated using Poisson regression models, and maternal age, high-risk pregnancy conditions, and numbers of previous pregnancies and births were employed to adjust. Number and percentage or median and interquartile range were used to describe baseline characteristics. The Chi-Square statistic and Mann-Whitney U-test were used for the comparison of categorical variables and continuous variables respectively [11]. Subjects for comparison of the results of clinical laboratory tests were matched by propensity score. The matching parameters were maternal age, high-risk pregnancy conditions, and number of previous pregnancies and births. The results of the clinical laboratory tests are presented in violin plot form. Mann-Whitney U test was used for intergroup statistical comparisons. Statistical analyses were conducted by R software (version 4.2.2) and GraphPad Prism (version 9.5.1).

## Results

After excluding 15, 8, and 2 pregnant women due to previous SARS-CoV-2 infection, ectopic pregnancy, and opting for abortion for foetal abnormalities respectively, we enrolled 818 pregnant women. These women had undergone SARS-CoV-2 nucleic acid testing during hospitalization for giving birth or experiencing pregnancy outcomes between 7 December 2022, and 10 January 2023 (Figure S1). Of all the subjects, 276 (33.7%) were diagnosed with SARS-CoV-2 during childbirth. Pregnant women who were infected or uninfected with SARS-CoV-2 during childbirth showed similar baseline characteristics, with median ages of 31 and 32 years and median maternal weights of 68.0 kg and 67.5 kg, respectively. According to the investigators’ assessment, 77.5% (214/276) and 81.0% (439/542) of these women were considered high-risk pregnancies respectively. Furthermore, the median numbers of previous pregnancies and births in both groups were 2 and 0 respectively (Table S1). All subjects did not exhibit any symptoms or signs related to severe COVID-19 during the observation period.

### Maternal and neonatal outcomes of pregnant women with COVID-19

To evaluate the impact of SARS-CoV-2 infection during childbirth on maternal and neonatal outcomes, we selected specific events for investigation. These include a maternal hospital length of stay (HLOS) of seven or more days, stillbirth, admission to the neonatal intensive care unit (NICU), low Apgar score (indicating a newborn Apgar score of less than 7), premature birth, and miscarriage. We assessed the impact of SARS-CoV-2 by comparing the incidence rate of outcomes above between the two groups of pregnant women. In our comparison, we adjusted the HR to correct for maternal age, conditions indicative of a high-risk pregnancy, and numbers of previous pregnancies and births.

**Figure 1.**
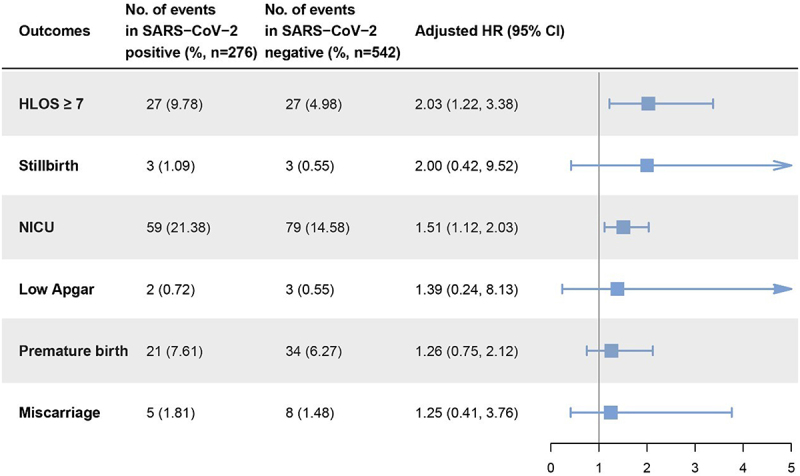
Maternal and perinatal outcomes among pregnant women with and without SARS-CoV-2 infection.

Among the outcomes, HLOS of seven or more days and admission to the NICU showed significant differences between groups. Specifically, the incidence of HLOS of seven or more days was 9.78% (27/276) in the SARS-CoV-2 positive group, compared to 4.98% (27/542) in the negative group, indicating that SARS-CoV-2 positive pregnant women had a 2.03-fold (95% CI, 1.22–3.38) increased risk of an extended HLOS. The incidence rate of NICU admission was relatively closer between groups, with 21.38% (59/276) in positive cases and 14.58% (79/542) in negative cases. Accordingly, newborns born to mothers infected with SARS-CoV-2 during childbirth had a 1.51-fold (95% CI, 1.12–2.03) increased risk of NICU admission compared to those born to non-infected mothers. The difference in the incidence of premature birth between the two groups was even more reduced, standing at 7.61% (21/276) and 6.27% (34/542) respectively, with an aHR of only 1.26 (95% CI, 0.75–2.12). Meanwhile, the incidence of stillbirth, low Apgar, and miscarriage were all less than 2% in both groups. On comparison, the aHRs were found to be 2.00 (95% CI, 0.42–9.52), 1.39 (95% CI, 0.24–8.13), and 1.25 (95% CI, 0.41–3.76) respectively (Figure 1).

Models adjusted for maternal age and high-risk pregnancy condition. Low Apgar refers to a newborn Apgar score of less than 7. HLOS, hospital length of stay. NICU, neonatal intensive care unit.

### Clinical laboratory testing of pregnant women with COVID-19

Among the pregnant women for whom routine laboratory test results at admission were available, we further matched 462 subjects based on infection with SARS-CoV-2 during childbirth, creating two equal groups of 231 subjects (Figure S1). After matching, there were no statistically significant differences between the two groups in terms of age, maternal weight, proportion of high-risk pregnancies, and so on (Table S2). In addition, we further evaluated the impact of SARS-CoV-2 infection on clinical laboratory test results using outcomes of HLOS ≥ 7 and NICU admission as subgroup variables.

**Figure 2.**
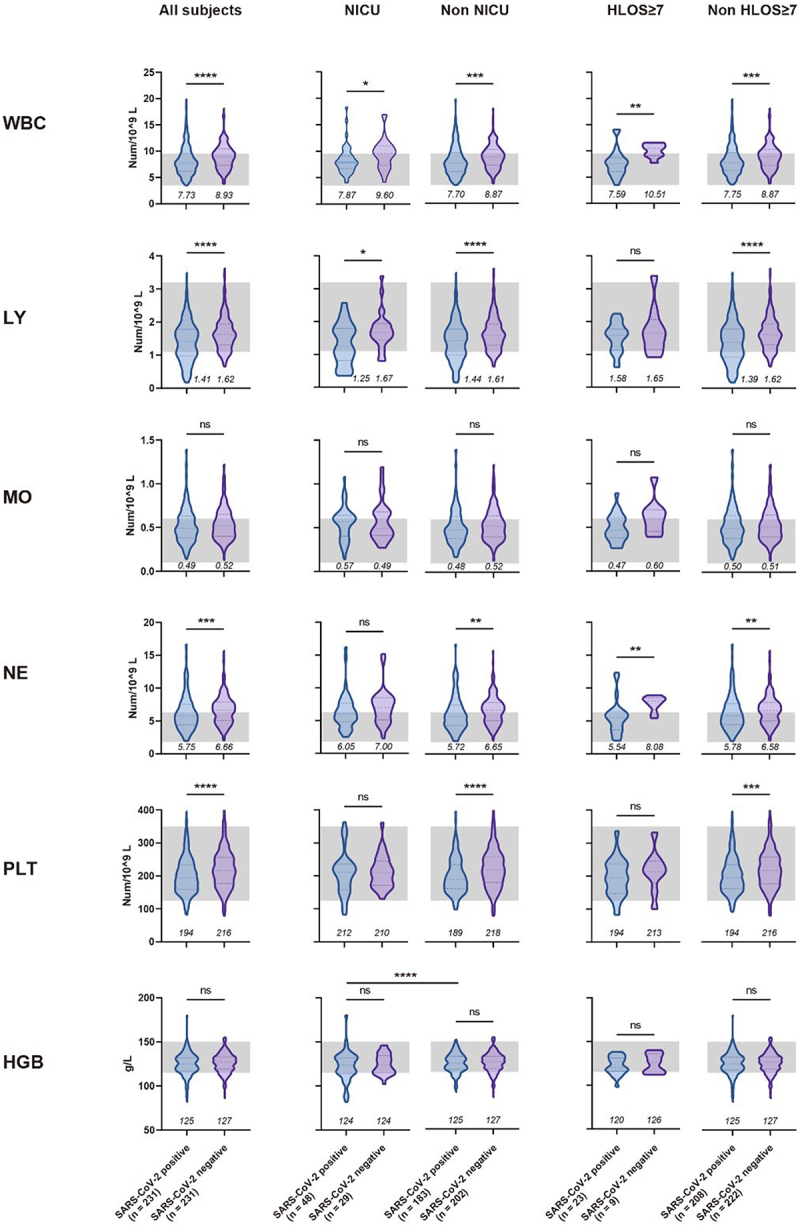
Comparison of blood routine test results upon admission for pregnant women, with or without SARS-CoV-2 infection, under different maternal and perinatal outcomes.

In the observation of routine blood tests (Figure 2), we found that pregnant women infected with SARS-CoV-2, like those with other viral infections, demonstrated a significant reduction in peripheral white blood cell (WBC) count (*p* < 0.0001). Additionally, within the WBC components, there was also a decline in lymphocytes (*p* < 0.0001), neutrophils (*p* < 0.001), and macrophages, although the decrease in macrophages was not significant. These alterations may be attributed to the recruitment of immune cells caused by respiratory inflammation [12]. The difference in WBC counts between the infected and non-infected groups was more pronounced in subjects with NICU admissions (1.73 vs 1.17) or HLOS ≥ 7 (2.92 vs 1.12). This suggests that the heightened degree of inflammation due to SARS-CoV-2 infection may increase the risk of adverse maternal and neonatal outcomes.

In the violin plot, the three dashed lines from top to bottom represent the upper, middle, and lower quartiles, respectively. The grey shaded area signifies the normal value range for this index in general adults. The numbers below the graph denote the median values. Mann-Whitney U test was used for statistical comparisons. Comparisons were also conducted between SARS-CoV-2 positive pregnant women with or without the results of NICU and HLOS ≥7 days, and only the significant *p* values were shown in the figures. ns, not significant. *, p < 0.05. **, p < 0.01. ***, p < 0.001. ****, p < 0.0001. HGB, haemoglobin. WBC, white blood cell. LY, lymphocyte. MO, macrophage. NE, neutrophil. PLT, platelet. HLOS, hospital length of stay. NICU, neonatal intensive care unit.

**Figure 3.**
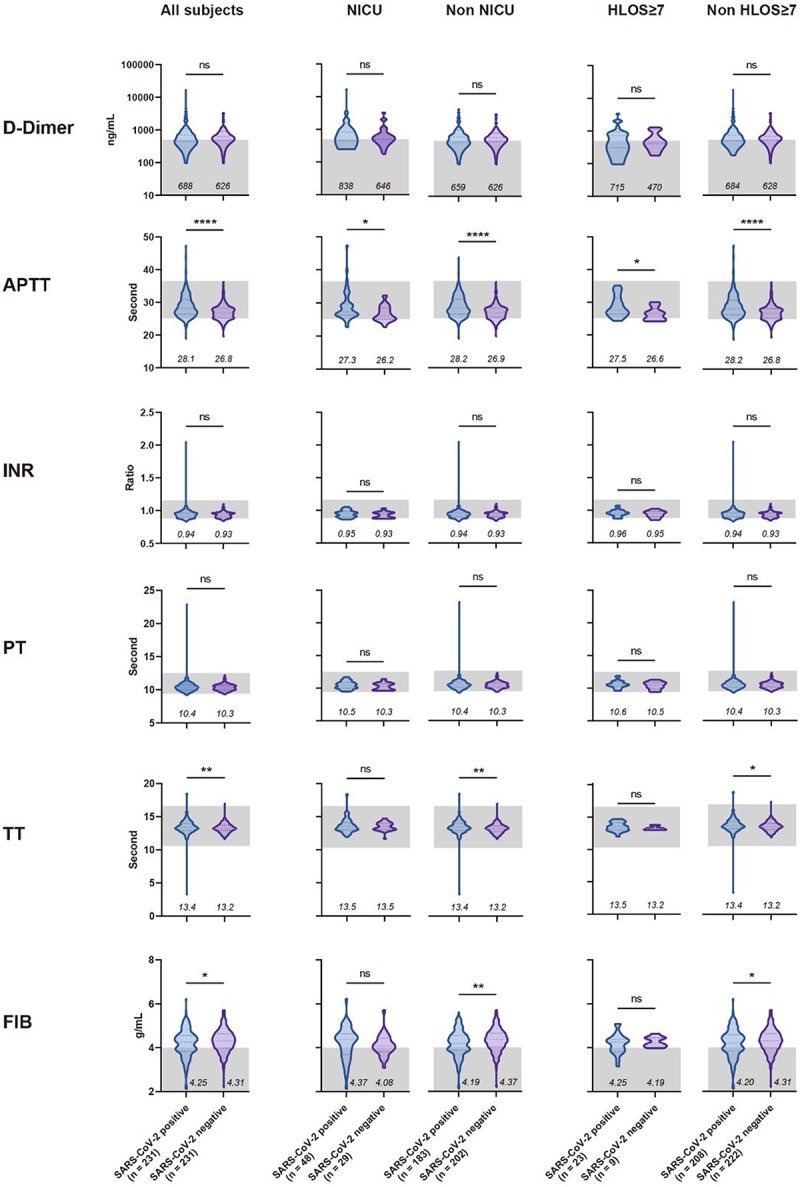
Comparison of coagulation test results upon admission for pregnant women, with or without SARS-CoV-2 infection, under different maternal and perinatal outcomes.

Among the coagulation-related indices, we observed a significant reduction (*p* < 0.001) in platelet count (Figure 2), along with minor prolongation in activated partial thromboplastin time (*p* < 0.0001) and thrombin time (*p* < 0.01) (Figure 3), among participants infected with SARS-CoV-2. This could be attributed to the fact that COVID-19 tends to cause more coagulation abnormalities, rather than a hypercoagulable state [13], which may be a result of vascular and endothelial damage caused by SARS-CoV-2 [14]. It is worth noting that among the subjects who experienced outcomes of NICU admission and HLOS ≥ 7, the difference between positive and negative in some coagulation indices was reduced or even reversed. For instance, in the NICU and HLOS ≥ 7 subgroups, fibrinogen in the positive group was higher than negative, while the trend was the opposite in the non-NICU and non-HLOS ≥7 subgroups. This might suggest that other factors driving adverse maternal and neonatal outcomes could potentially mask the impact of COVID-19 on coagulation function.

In the violin plot, the three dashed lines from top to bottom represent the upper, middle, and lower quartiles, respectively. The grey shaded area signifies the normal value range for this index in general adults. The numbers below the graph denote the median values. Mann-Whitney U test was used for statistical comparisons. Comparisons were also conducted between SARS-CoV-2 positive pregnant women with or without the results of NICU and HLOS ≥7 days, and only the significant *p* values were shown in the figures. ns, not significant. *, p < 0.05. **, p < 0.01. ****, p < 0.0001. APTT, activated partial thromboplastin time. INR, international normalized ratio. PT, prothrombin time. TT, thrombin time. FIB, fibrinogen. HLOS, hospital length of stay. NICU, neonatal intensive care unit.

**Figure 4.**
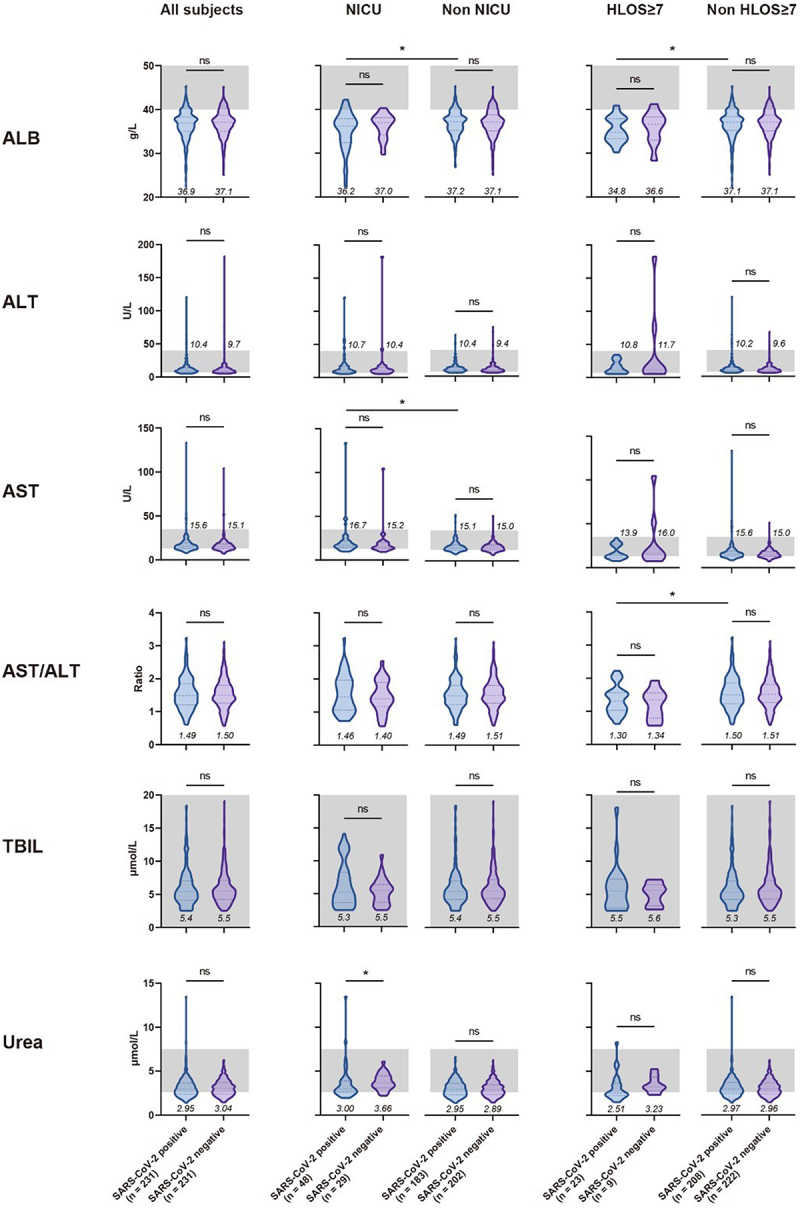
Comparison of metabolic test results upon admission for pregnant women, with or without SARS-CoV-2 infection, under different maternal and perinatal outcomes.

In the comparative analysis of metabolic test results, almost all indices variations were not significant (Figure 4), most likely due to the fact that the subjects were non-severe COVID-19 cases and the disease had not yet imposed considerable strain on hepatic and renal functions. In addition, given all the subjects were in the late stages of pregnancy, their albumin levels were relatively lower compared to non-pregnant standards, and among positive subjects, a significant decrease in albumin (*p* < 0.05) was found amongst individuals with NICU admissions or HLOS ≥7 compared to those without. Coupling this with the results of haemoglobin levels in the two outcome groups (Figure 2), we postulate a possible correlation between the nutritional status of pregnant women and the risk of adverse maternal and neonatal outcomes among positive pregnant women.

In the violin plot, the three dashed lines from top to bottom represent the upper, middle, and lower quartiles, respectively. The grey shaded area signifies the normal value range for this index in general adults. The numbers below the graph denote the median values. Mann-Whitney U test was used for statistical comparisons. Comparisons were also conducted between SARS-CoV-2 positive pregnant women with or without the results of NICU and HLOS ≥7 days, and only the significant *p* values were shown in the figures. ns, not significant. *, p < 0.05. ALB, albumin. ALT, alanine aminotransferase. AST, aspartate aminotransferase. TBIL, total bilirubin. HLOS, hospital length of stay. NICU, neonatal intensive care unit.

**Figure 5.**
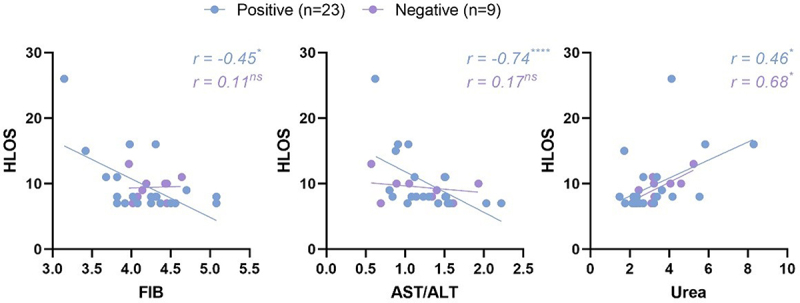
In women with an HLOS of seven days or more, some laboratory test indices showed correlations with HLOS.

Within the subjects having HLOS of 7 or more days, some indices revealed intriguing correlations with HLOS (Figure 5). In the positive group, both fibrinogen and ratio of aspartate aminotransferase and alanine aminotransferase (AST/ALT) depicted a substantial negative correlation with HLOS, with correlation coefficients of −0.45 (*p* < 0.05) and −0.74 (*p* < 0.0001) respectively, while such correlation was not observed in the negative group. This phenomenon, to a certain degree, signifies the impact of pronounced coagulation disorders and hepatitis caused by COVID-19 on extending the HLOS for pregnant women. The negative correlation between AST/ALT and HLOS also indicates that the hepatitis caused by mild COVID-19 in pregnant women prominently reflects the characteristics of early acute hepatitis. Furthermore, urea exhibited a significant positive correlation with HLOS in both positive and negative groups, with correlation coefficients of 0.46 (*p* < 0.05) and 0.68 (*p* < 0.05) respectively, suggesting the association of renal dysfunction with adverse maternal outcomes.

The symbol following the correlation coefficient represents the P-value, used to statistically test the significance of the correlation coefficient. P-values resulted from a two-tailed test for the Spearman rank correlation coefficient. FIB, fibrinogen. ALT, alanine aminotransferase. AST, aspartate aminotransferase. HLOS, hospital length of stay. ns, not significant. *, *p* < 0.05. ****, *p* < 0.0001.

## Discussion

The focus of COVID-19 response efforts has increasingly shifted towards high-risk populations in the post-pandemic era. Given their critical role in nurturing the next generation, the health outcomes of pregnant women following COVID-19 infection have naturally emerged as a primary concern.

During the COVID-19 pandemic, particularly in the early Omicron phase, there was a notable increase in mortality and morbidity among pregnant women [15,16]. This situation has significantly heightened concerns regarding the health outcomes of pregnant women due to COVID-19 infection. To assess the impact of COVID-19 on maternal and neonatal outcomes and clinical laboratory testing under the BA.5.2/BF.7 surge, we enrolled 818 pregnant women who were grouped based on their SARS-CoV-2 nucleic acid test results during childbirth, within one month from December 2022 to January 2023. Comparing the occurrence of maternal and neonatal outcomes, we found that non-severe COVID-19 still significantly increased the risk of extended HLOS and NICU admission. However, there was no significant impact on the occurrence of stillbirth, low Apgar, premature birth, and miscarriage. Our findings regarding premature birth, low Apgar score, and stillbirth align with the studies conducted by Mia Ahlberg [17], and Kate R Woodworth [18]. However, Shu Qin Wei et al.“s meta-analysis found a significant impact of COVID-19 on premature birth and stillbirth [19]. A Canadian study revealed that COVID-19 notably elevates the risk of preterm birth while having an insignificant effect on stillbirth rates [20]. We posit that discrepancies in findings between this study and others can be attributed to the following factors. First, the limited sample size constrained our ability to gather enough comparable events. Second, the exclusive inclusion of non-severe COVID-19 cases means that the observed antiviral response among pregnant women might have been minimal. An immunology study focusing exclusively on pregnant women with mild COVID-19 identified restrained immune activation at the maternal-fetal interface, absent the cytokine storm typically triggered by an overproduction of inflammatory factors [21]. Another study involving pregnant women with severe COVID-19 revealed a pronounced enhancement in cytokine secretion. Additionally, it found that the maternal-fetal immune interaction at the interface was disrupted in those infected with SARS-CoV-2 [22]. These immunological insights help elucidate the observed lack of a notably higher incidence of severe clinical outcomes, such as stillbirth, in pregnant women with COVID-19 in comparison to the non-infected group. Third, the incidence of grave clinical outcomes, including stillbirths, may be attributed to the quality of clinical care afforded to expectant mothers. Research conducted by Barbara and colleagues [16] demonstrated that the notable escalation in stillbirth risk throughout the pandemic was confined to low- and middle-income countries, with no similar trends observed in high-income nations. Moreover, race and ethnic disparities among participants may result in different outcomes. This study was confined to the evaluation of East Asians, in contrast to Elisabeth et al.”s research, which predominantly involved Caucasian subjects. They also mentioned that a heightened risk of preterm birth due to SARS-CoV-2 infection was noted in studies predominantly involving Caucasian populations, such as in the UK and Nordic countries [20].

In conclusion, we found that even non-severe COVID-19 during the BA.5.2/BF.7 surge was associated with approximately a 2-fold increase in HLOS of seven days or more and a 1.5-fold increase in the risk of NICU admission for newborns. This suggests that protective measures against COVID-19 still have a positive effect on reducing the risk of adverse maternal and neonatal outcomes for pregnant women.

We further inspected the effect of COVID-19 on the results from admitted clinical laboratory tests based on 462 matched pregnant women. It is plausible that due to the recruitment of respiratory inflammation [12,23], we detected a significant reduction in WBC and its components within the SARS-CoV-2 positive group. Additionally, in subgroups where HLOS ≥ 7 and NICU admitted occurred, the disparities of indices between positive and negative broadened. This partially explains that COVID-19 may escalate the risk of adverse maternal and neonatal outcomes through inflammation-driven processes. This observation aligns with trends identified in research involving placental samples and umbilical cord blood [21,22]. The reduction in lymphocytes, as shown in previous studies [24–26], mainly reflects a decrease in the number of T cells, including specific cell subsets such as Th1 and Tc17, which play crucial roles in mediating pro-inflammatory responses under both healthy and diseased conditions [22,27]. During pregnancy, these subsets also participate in establishing and maintaining maternal-foetal tolerance [28–30]. Downregulation of related cells, as induced by COVID-19, may impair this tolerance mechanism, potentially increasing the risk of immune-related conditions such as atopic dermatitis in the offspring [31].

Consistent with trends demonstrated in previous studies concerning non-pregnant populations [32–34], our research also found that pregnant women infected with SARS-CoV-2, as compared to those not infected, presented a propensity towards coagulation disorders. These disorders specifically manifested as a reduction in platelet counts, as well as prolongation of activated partial thromboplastin time and thrombin time. Inter-group differences in metabolic test indices were not significant. However, we observed that SARS-CoV-2 infection appeared to amplify certain nutrition-related differentials (such as albumin and haemoglobin) between groups that experienced adverse maternal and neonatal outcomes and those that did not. This suggests that additional nutritional status checks might be necessary for pregnant women infected with SARS-CoV-2 to prevent adverse maternal and neonatal outcomes. Lastly, in our correlation analysis between certain index values and the HLOS, we noted a distinct negative correlation between fibrinogen and AST/ALT with HLOS in the positive group, which was not observed in the negative group. This further indicates the prognostic value of clinical tests for pregnant women infected with SARS-CoV-2.

However, several limitations should be noted for our study. First, due to the lack of effective means to obtain vaccination information for our study subjects, we could only speculate about their COVID-19 vaccination status based on public information. With the average number of vaccines administered per person in China exceeding 2.4 and over 90% of the population having been vaccinated [35,36]—and considering that main vaccination campaigns took place prior to 2022 [37]—we infer that the subjects in our study likely have a high vaccination rate. Second, we failed to conduct sequencing analyses on subjects infected with SARS-CoV-2 to ascertain their infection with the BA.5.2/BF.7 variants. Besides, the limited sample size may have precluded the observation of significant clinical outcomes, such as morbidities in the respiratory and nervous systems. Additionally, the absence of severe COVID-19 cases or fatalities in our study could have restricted the clinical significance of this study regarding severe COVID-19 outcomes in pregnant women.

In summary, by studying pregnant women delivering during the BA.5.2/BF.7 surge period, we discovered that COVID-19 significantly increased the risk of having an HLOS of seven days or more and neonatal admission to the NICU. Through the analysis of results from clinical laboratory tests conducted at admission, we observed a series of pathological or physiological patterns related to immunity, coagulation, and metabolism as indicated by various indices. Our research provides valuable guidance for clinical practice with pregnant women infected with the recently emerged Omicron subvariants.

## Supplementary Material

Supplemental Material

## Data Availability

the Data generated during the study is available at repository “figshare” at https://figshare.com/articles/dataset/COVID-19_in_pregnant_women_original_data_csv/25764261 and lastly the reference number [10.6084/m9.figshare.25764261].
