# Supplementary material for: Maternal and neonatal outcomes and clinical laboratory testing of pregnant women with COVID-19 during the BA.5.2/BF.7 surge
Source: Virulence. 2024 May 27;15(1):2360130. doi: 10.1080/21505594.2024.2360130 (PMC11152110; doi:10.1080/21505594.2024.2360130)
Supplement: Supplemental Material [file KVIR_A_2360130_SM8474.docx]

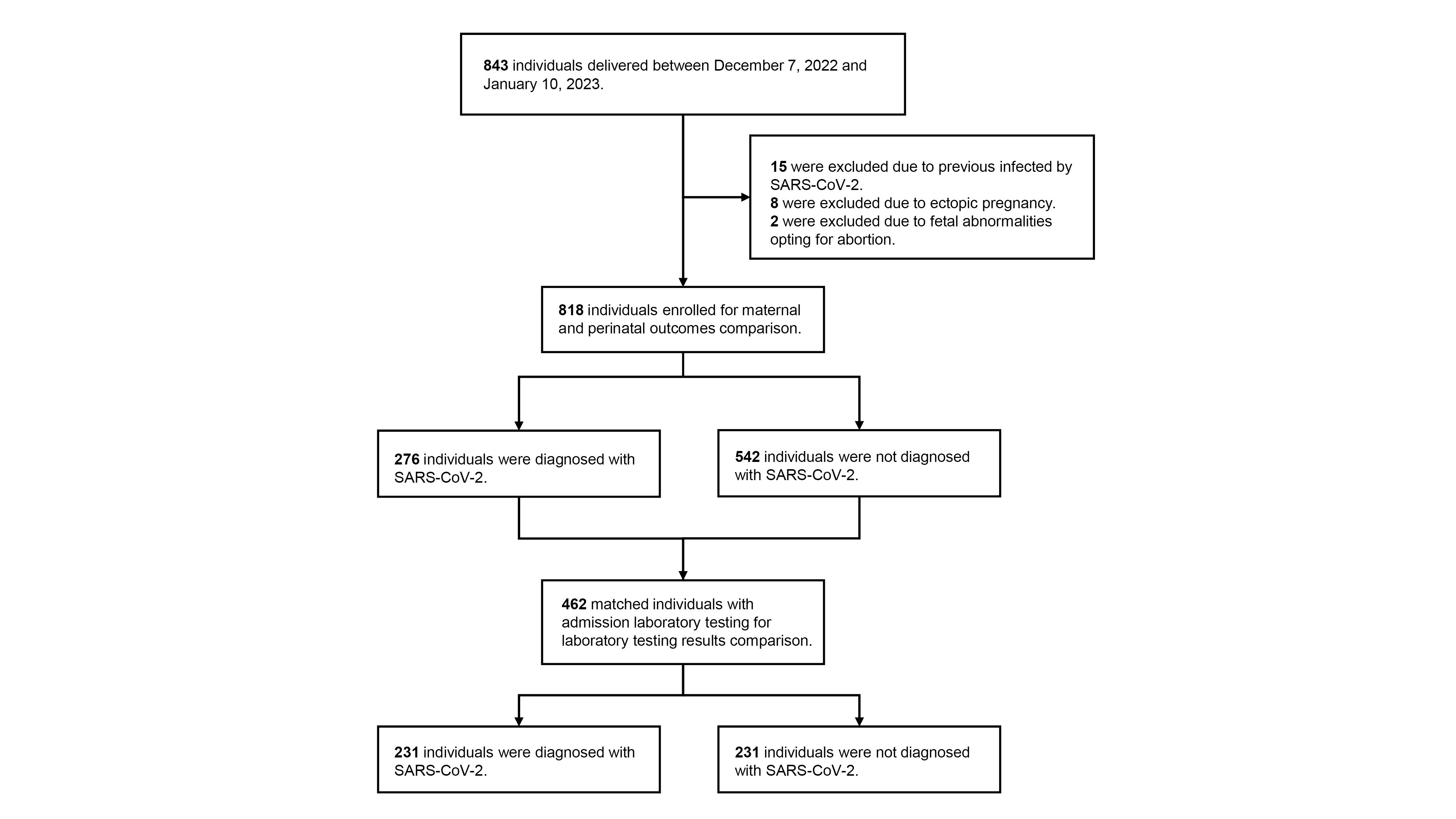


**Figure S1. Flow chart describing the participants' enrollment.**

Supplemental Table S1. **Characteristics of all the participants.**

|  | **Pregnant women infected with SARS-CoV-2  (n=276) median (IQR) or n (%)** | **Pregnant women not infected with SARS-CoV-2  (n=542) median (IQR) or n (%)** |
| --- | --- | --- |
| **Age** | 31 (6.0) | 32 (5.0) |
| **Maternal weight (kg)** | 68.0 (11.7) | 67.5 (12.0) |
| **Previous pregnancy** | 2 (2.0) | 2 (2.0) |
| **Previous birth** | 0 (1.0) | 0 (1.0) |
| **High-risk pregnancy** | 214 (77.5) | 439 (81.0) |
| Age beyond 15-35 | 62 (22.5) | 123 (22.7) |
| Wight beyond 40-85 kg | 17 (6.2) | 23 (4.2) |
| History of abnormal pregnancy | 101 (36.6) | 226 (41.7) |
| Medical conditions of pregnancy (including diabetes, hypertension, hypothyroidism, and gestational cholestasis) | 102 (37.0) | 219 (40.4) |
| Poor delivery conditions (including pelvic abnormalities, multiple births, and abnormal fetal position, macrosomia) | 31 (11.2) | 40 (7.4) |
| Cicatricial uterus | 39 (14.1) | 102 (18.8) |
| Excess or low amniotic fluid | 9 (3.3) | 19 (3.5) |
| In vitro fertilization or embryo transfer | 21 (7.6) | 38 (7.0) |
| Abnormal reproductive structures | 22 (8.0) | 44 (8.1) |
| Congenital anomalies or fetal growth retardation | 0 (0) | 3 (0.6) |
| Thalassemia | 14 (5.1) | 28 (5.2) |
| Placenta praevia, placenta abruptio, or hypofunction | 4 (14) | 11 (2.0) |

IQR, interquartile range.

Supplemental Table S2. **Characteristics of the matched participants for the comparison of laboratory testing.**

|  | **Pregnant women infected with SARS-CoV-2  (n=231) median (IQR) or n (%)** | **Pregnant women not infected with SARS-CoV-2  (n=231) median (IQR) or n (%)** | **P-values** |
| --- | --- | --- | --- |
| **Age** | 31 (6.0) | 31 (6.0) | 0.9922 |
| **Maternal weight (kg)** | 68.0 (12.0) | 67.5 (12.0) | 0.2256 |
| **Previous pregnancy** | 2 (2.0) | 2 (2.0) | 0.5305 |
| **Previous birth** | 0 (1.0) | 0 (1.0) | 0.5377 |
| **High-risk pregnancy** | 180 (77.5) | 173 (81.0) | 0.5109 |

The Chi-Square statistic was used for categorical variables. Continuous variables were compared by using a two-sided Mann-Whitney U-test. IQR, interquartile range.
